# Foxp3^+^ T reg cells control psoriasiform inflammation by restraining an IFN-I–driven CD8^+^ T cell response

**DOI:** 10.1084/jem.20172094

**Published:** 2018-08-06

**Authors:** Krista Stockenhuber, Ahmed N. Hegazy, Nathaniel R. West, Nicholas E. Ilott, Alexander Stockenhuber, Samuel J. Bullers, Emily E. Thornton, Isabelle C. Arnold, Andrea Tucci, Herman Waldmann, Graham S. Ogg, Fiona Powrie

**Affiliations:** 1 Kennedy Institute of Rheumatology, University of Oxford, Oxford, UK; 2 Translational Gastroenterology Unit, Nuffield Department of Clinical Medicine, University of Oxford, Oxford, UK; 3 MRC Human Immunology Unit, Weatherall Institute of Molecular Medicine, Radcliffe Department of Medicine, University of Oxford, Oxford, UK; 4 Wellcome Trust Centre for Human Genetics, Radcliffe Department of Medicine, University of Oxford, Oxford, UK; 5 Sir William Dunn School of Pathology, University of Oxford, Oxford, UK

## Abstract

Psoriasis is a complex inflammatory skin disease affecting ∼3% of the population worldwide. Although type I interferons (IFN-I) are thought to be involved in its pathogenesis, the details of this relationship remain elusive. Here we show that in a murine model of imiquimod-driven psoriatic skin inflammation, Foxp3^+^ regulatory T cells (T reg cells) control inflammation severity by restraining IFN-I. Depletion of T reg cells induces IFN-I and IFN-stimulated gene expression, and leads to accumulation of CD8^+^ T cells in lesional skin. Mononuclear phagocytes (MNPs) were the source of IFN-I, and their depletion reversed the effect of T reg cell depletion. Blockade of IFN-I signaling abolished CD8^+^ T cell infiltration and excess inflammation in the skin of T reg cell–depleted mice. Depletion of CD8^+^ T cells attenuated pathology, confirming their role as critical effector cells downstream of IFN-I. Our results describe an unexpected role for T reg cells in restraint of an MNP–IFN-I–driven CD8^+^ T cell response during psoriasiform skin inflammation. These findings highlight a pathway with potential relevance for the treatment of early-stage disease.

## Abstract (precis)

Stockenhuber et al. demonstrate that Foxp3^+^ regulatory T cells control psoriasiform skin inflammation by restraining a type I interferon (IFN-I)–driven CD8^+^ T cell response. The absence of T reg cells led to exacerbated inflammation, infiltration of CD8^+^ T cells to the epidermis, and an IFN-I gene signature in the tissue.

## Graphical Abstract

**Figure:**
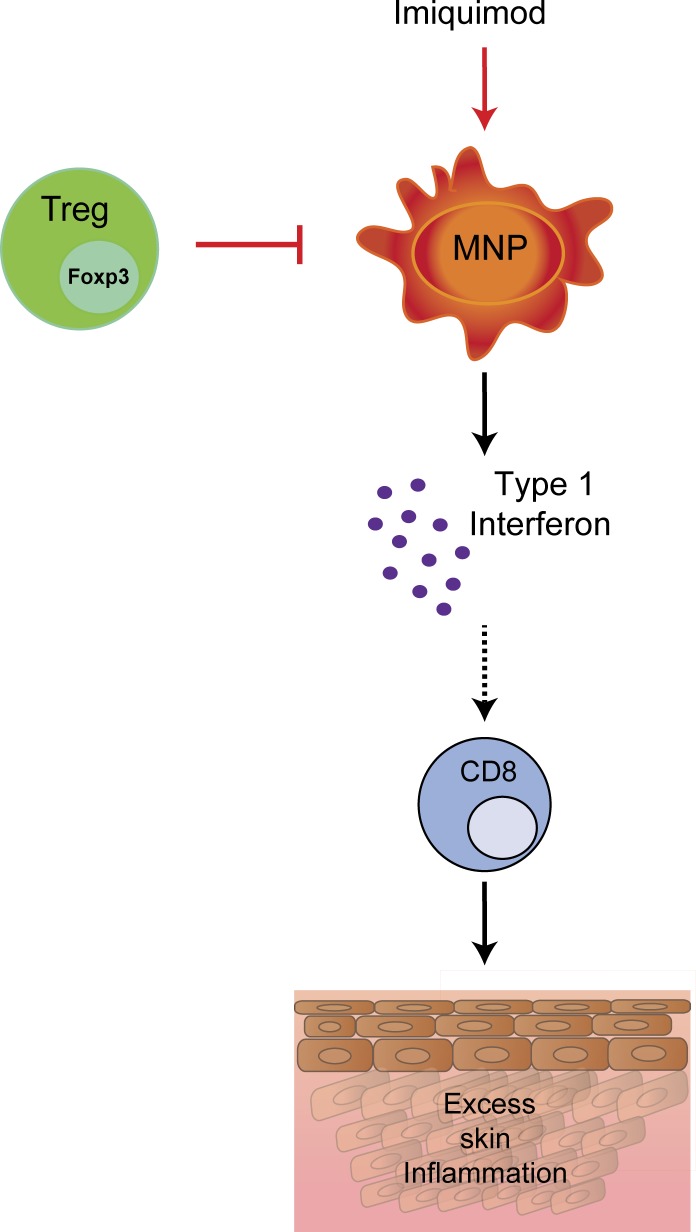

## Introduction

Psoriasis is a chronic inflammatory skin disease that affects 3% of the world’s population. It follows a relapsing–remitting pattern and carries a high disease burden (Nestle et al., 2009; Lowes et al., 2014). The most common form, known as plaque psoriasis, or psoriasis vulgaris, features widespread erythematous plaques with adherent scales that persist for weeks to months, and affected patients often require life-long treatment (Nestle et al., 2009; Lowes et al., 2014).

Psoriasis pathogenesis is complex and poorly understood, due in part to its chronicity, which makes the identification of pathways involved in disease initiation difficult. In particular, the immunopathology and regulation of events leading to acute plaque formation remain ill defined. Several studies have implicated type I interferon (IFN-I) in the early pathogenesis of psoriasis, but their precise role remains elusive (Kariniemi, 1977; Grine et al., 2015). In a xenograft murine model, blockade of IFN-I prevented plaque formation (Nestle et al., 2005). Notably, patients undergoing interferon therapy can develop or aggravate psoriasis (La Mantia and Capsoni, 2010; Afshar et al., 2013). Furthermore, variants in genes involved in viral sensing and IFN-I induction are associated with risk of psoriasis (Bijlmakers et al., 2011; Tsoi et al., 2012). A clinical trial of IFN-I blockade in patients with long-standing psoriasis failed to show an effect; however, the study did not examine early disease processes (Bissonnette et al., 2010).

IFN-I can promote CD8^+^ T cell responses, and CD8^+^ T cells are known to accumulate in the epidermis of psoriatic skin (Hammar et al., 1984; Cheuk et al., 2014). Depletion of CD8^+^ T cells in an IFN-I–dependent model of psoriasis inhibited inflammation and plaque formation, indicating a role for CD8^+^ T cells in that model (Di Meglio et al., 2016).

Foxp3^+^ regulatory T cells (T reg cells) comprise a large proportion of skin-resident CD4^+^ T cells in mice and humans (Wohlfert et al., 2011; Sanchez Rodriguez et al., 2014; Scharschmidt et al., 2015). Deficiencies in T reg cell function have been postulated to underpin psoriasis (Sugiyama et al., 2005). Indeed, in patients with immunodysregulation polyendocrinopathy enteropathy X-linked (IPEX) syndrome due to *FOXP3* mutations, or in patients with IPEX-like diseases caused by mutations in related genes (Halabi-Tawil et al., 2009; Goudy et al., 2013), psoriasis-like skin inflammation and epidermal infiltration by CD8^+^ T cells are some of the earliest and most severe features (Halabi-Tawil et al., 2009; Goudy et al., 2013). Nevertheless, the role of T reg cells in psoriasis is incompletely understood (Belkaid and Tamoutounour, 2016; Ali and Rosenblum, 2017).

To dissect the function of Foxp3^+^ T reg cells during psoriasis development, we depleted T reg cells in the imiquimod (IMQ)-induced model of psoriasiform skin inflammation. IMQ is a potent TLR7/8 ligand that is used clinically to treat neoplastic skin lesions. It can lead to deterioration of psoriasis in patients with well-controlled disease and trigger psoriasis in previously unaffected individuals (Gilliet et al., 2004; Fanti et al., 2006; Rajan and Langtry, 2006). To enable specific deletion of T reg cells in the IMQ model, we used the Foxp3^hCD2^ mouse, in which the mouse *Foxp3* promoter controls expression of human CD2. This enables both reporting of Foxp3^+^ T reg cells and their depletion using an anti-hCD2 monoclonal antibody (Komatsu et al., 2009; Kendal et al., 2011). Strikingly, T reg cell depletion promoted IFN-I production by mononuclear phagocytes (MNPs), which drove an epidermal CD8^+^ T cell response that exacerbated skin inflammation. These findings demonstrate that T reg cells can control the severity and quality of psoriasis-like inflammation by regulating the IFN-I response.

## Results and discussion

### Foxp3^+^ T reg cells accumulate in psoriasiform skin and control inflammation severity

T reg cells from the blood of psoriatic patients are impaired in their capacity to inhibit effector T cells (T eff cells) in vitro (Sugiyama et al., 2005), and lesional skin of psoriatic patients exhibits an abnormally low T reg/T eff cell ratio (Keijsers et al., 2013). Furthermore, newly formed “acute” plaques are reported to be T reg cell deficient (Yun et al., 2010). Based on these studies, we hypothesized that T reg cells play a critical role in psoriasiform inflammation and employed the IMQ model to identify the functional consequences of T reg cell depletion during plaque formation.

**Figure 1.**
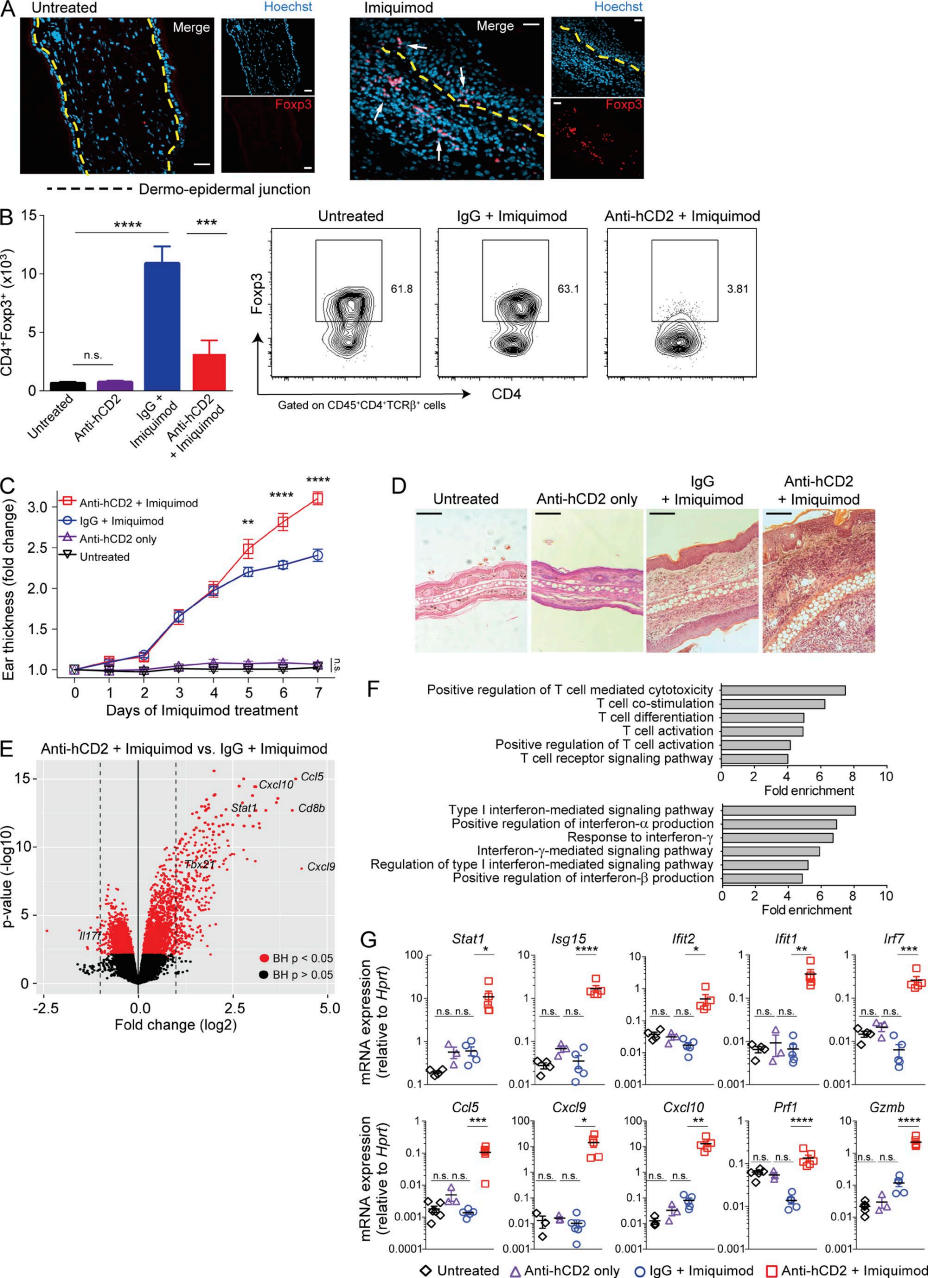
**Foxp3^+^ T reg cells accumulate in psoriasiform inflammation and control the inflammatory response in the skin. (A)** Representative immunofluorescence staining of Foxp3^+^ cells (examples indicated by arrows) in untreated and IMQ-treated ear skin of B6 wild type mice. Dashed lines indicate the dermo-epidermal junction. Scale bars, 20 µm. **(B)** Flow cytometry analysis of Foxp3^+^ T reg cells in ear skin from Foxp3^hCD2^ mice treated as indicated. Left, T reg cell number; right, representative flow cytometry plots. **(C)** Thickness of ear skin over the course of treatment with IMQ. **(D)** Representative H&E staining of ear tissue cross sections. Scale bars, 200 µm. Data in panels A–D are representative of one of two or more experiments with *n* ≥ 4 mice per group. **(E)** Change in gene expression in anti-hCD2 + IMQ– versus IgG + IMQ–treated skin (*n* = 3 per group, one experiment). **(F)** Differentially regulated GO pathways in anti-hCD2 + IMQ–treated skin, including T cell– (top) and interferon-related pathways (bottom); all indicated pathways were significant with false discovery rates <0.05 based on hypergeometric test (*n* = 3 per group, one experiment). **(G)** qPCR analysis confirming mRNA levels of interferon-stimulated genes in untreated, anti-hCD2 only–, IgG + IMQ–, or anti-hCD2 + IMQ–treated skin (representative of one of two or more experiments with *n* ≥ 3 mice per group). Error bars: means ± SEM. Statistics: one-way (B) and two-way ANOVA (C) with post-hoc test; empirical Bayes method with Benjamini-Hochberg (BH) adjusted P values (E); Mann-Whitney *U* test (G). *P = 0.01–0.05, **P = 0.001–0.01, ***P = 0.0001–0.001, ****P < 0.0001, n.s., not significant.

**Figure 2.**
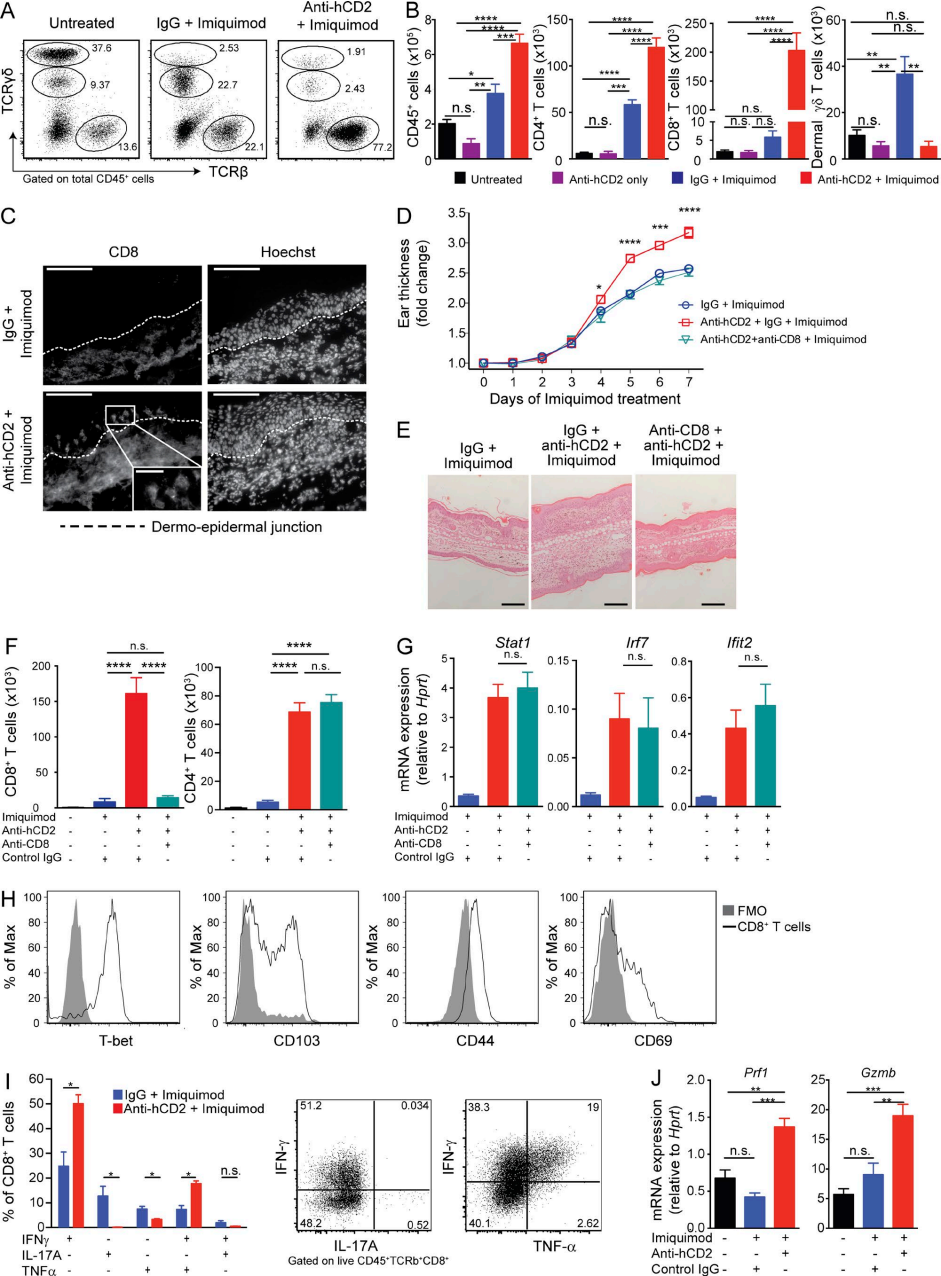
**CD8^+^ T cells are the driving effector population controlled by T reg cells in psoriasiform skin inflammation. (A and B)** Flow cytometry analysis of T cell subsets in mouse ear skin. Frequency of TCRβ^+^, TCRγδ^int^, and TCRγδ^high^ cells among total CD45^+^ cells (A) and absolute numbers of cells extracted from skin (B). **(C)** Representative immunofluorescence staining of CD8^+^ T cells in ear skin. Dashed lines indicate the dermo-epidermal junction. Scale bars, 100 µm; inset, 25 µm. **(D)** Thickness of ear skin over the course of IMQ treatment. **(E)** Representative H&E staining after 7 d of IMQ treatment. Scale bars, 200 µm. **(F)** Total CD8^+^ and CD4^+^ T cells extracted from skin, determined by flow cytometry. **(G)** qPCR analysis confirming mRNA levels of interferon response genes in IMQ-treated skin. **(H)** Flow cytometry analysis of CD8^+^ T cells isolated from anti-hCD2 + IMQ–treated ear skin. FMO, fluorescence minus one control. **(I)** Frequency of IFN-γ, IL-17A, and TNF-α expression by CD8^+^ T cells isolated from anti-hCD2 + IMQ– and IgG + IMQ–treated ear skin assessed by flow cytometry after stimulation (PMA/ionomycin for 4 h + BFA) and representative FACS plots for anti-hCD2 + IMQ–treated group. **(J)** qPCR analysis of FACS-sorted skin CD8^+^ T cells. Error bars: means ± SEM. Statistics: one-way (B, F, G, and J) and two-way ANOVA (D) with post-hoc test, Mann-Whitney *U* test (I). Data are representative of one of three (A–F and H) and one of two (F and I) experiments with *n* ≥ 2 (C and J) or *n* ≥ 3 mice per group (A, B, and D–I). *P = 0.01–0.05, **P = 0.001–0.01, ***P = 0.0001–0.001, ****P < 0.0001, n.s., not significant.

To explore the role of T reg cells in the IMQ model, we first used immunohistochemistry to detect Foxp3^+^ T reg cells in untreated and IMQ-treated ear skin of wild type mice. This revealed a notable increase in the numbers of T reg cells in the dermis and epidermis following IMQ treatment (Fig. 1 A). To determine their functional importance, we depleted T reg cells systemically by administering a depleting anti-hCD2 antibody to Foxp3^hCD2^ reporter mice 2 d before starting topical IMQ treatment for 7 consecutive days. Flow cytometry analysis demonstrated that T reg cells were significantly increased in IMQ-treated skin and that T reg cell depletion was highly efficient (Fig. 1 B). As previously described, administration of anti-hCD2 alone did not elicit significant inflammatory responses (Komatsu et al., 2009; Kendal et al., 2011; Fig. 1, C and D; Fig. S1 A; and Fig. 2 B).

Over the course of IMQ treatment, the average ear thickness increased 2–2.5-fold in mice treated with a nontargeting control antibody and over threefold in mice with T reg cell depletion (Fig. 1 C). Histological analysis of T reg cell–depleted skin revealed an increase in dermal thickness and accumulation of inflammatory cells in the dermal and epidermal skin layers (Fig. 1 D). These findings suggest that Foxp3^+^ T reg cells are important in controlling the severity of skin inflammation.

Dermal γδ T cells and T helper type 17 (Th17) cytokines are critical for pathogenesis in the conventional IMQ model (Cai et al., 2011; Pantelyushin et al., 2012). However, these factors were not exacerbated by T reg cell depletion in IMQ-treated mice (Fig. S1 C). To clarify the role of T reg cells during IMQ treatment, we performed transcriptomic analysis of whole skin tissue from IMQ-treated mice with or without T reg cell depletion. 285 genes were differentially expressed (272 up- and 13 down-regulated) in the T reg cell–depleted, IMQ-treated skin (Fig. 1 E) relative to IMQ-treated skin with normal T reg cell abundance. Based on gene ontology (GO) analysis, pathways associated with T cell activation, migration, and cytotoxicity, as well as interferon signaling, were strongly associated with T reg cell–depleted inflamed skin (Fig. 1 F). IFN-I–mediated signaling was the most highly enriched GO term. Quantitative RT-PCR analysis confirmed the up-regulation of interferon-stimulated genes in T reg cell–depleted psoriasiform skin (Fig. 1 G). Importantly, this gene signature was not evident in T reg cell–depleted uninflamed skin, as evidenced by similar amounts of IFN-response genes in skin tissue of untreated as well as anti-hCD2 only–treated animals (Fig. 1 G). Together, these data demonstrate that T reg cells accumulate in the skin during IMQ-induced inflammation, dampen disease severity, and inhibit the IFN-I response.

### CD8^+^ T cells are a critical effector population controlled by T reg cells in psoriasiform skin inflammation

Our transcriptomic analysis revealed strong up-regulation of several chemokines associated with CD8^+^ T cell recruitment in T reg cell–deficient skin, such as *Ccl5*, *Cxcl9*, and *Cxcl10*. Up-regulation of *Gzmb* (granzyme-B) and *Prf1* (perforin) further suggested that T reg cell deficiency triggers an influx of cytotoxic CD8^+^ T cell populations (Fig. 1 G). Unlike human psoriasis, the majority of T eff cells in IMQ-treated mouse skin are γδ T cells (van der Fits et al., 2009; Pantelyushin et al., 2012). Therefore, we next examined T eff influx in our T reg cell–depleted IMQ model. Flow cytometry analysis of CD45^+^ cells in IMQ-treated skin confirmed the expansion of γδ^dim^ T cells in the control IgG group, while TCRβ-expressing cells were the dominant T cell population in T reg cell–depleted inflamed skin (Fig. 2 A). Total skin CD45^+^ cell numbers were significantly higher in the T reg cell–depleted group than controls (Fig. 2 B). In T reg cell–sufficient, IMQ-treated mice, skin T cells were primarily dermal γδ T cells and CD4^+^ T cells (Fig. 2 B). By contrast, CD8^+^ T cells were the dominant T cell population in T reg cell–depleted skin. Whereas CD4^+^ T cells increased two- to fourfold in inflamed T reg cell–depleted skin compared with inflamed T reg cell–sufficient skin, the corresponding enrichment of CD8^+^ T cells was >25-fold (Fig. 2 B). The increase of dermal γδ T cells observed in T reg cell–sufficient, IMQ-treated skin was not observed in T reg cell–depleted inflammation (Fig. 2 B). Furthermore, a significantly lower proportion of γδ T cells produced IL-17A and IL-22 in T reg cell–deficient compared with T reg cell–sufficient IMQ-treated animals (Fig. S1, B and C). In the tissue, expression of *Il17a*, *Il17c*, and *Il17f* was significantly reduced between the T reg cell–deficient and –sufficient inflamed conditions (Fig. 1 E and Fig. S1 B). As in patients with psoriasis or IPEX-like syndromes, CD8^+^ T cells infiltrated the epidermis of IMQ- and anti-hCD2–treated mice (Fig. 2 C).

To determine the functional importance of CD8^+^ T cells in T reg cell–deficient mice, we depleted them using an anti-CD8 antibody, which reduced CD8^+^ T cell abundance to normal levels without affecting CD4^+^ T cell abundance (Fig. 2 F). Ear thickness and histology in mice with combined T reg cell and CD8^+^ T cell depletion were equivalent to that of T reg cell–sufficient, IMQ-treated animals (Fig. 2, D and E). Interestingly, interferon-stimulated gene expression remained unchanged despite CD8^+^ T cell depletion and reduced pathology, indicating that T reg cells potentially control a mechanism upstream of CD8^+^ T cells to prevent excess skin inflammation (Fig. 2 G). Skin-infiltrating CD8^+^ T cells expressed CD103, CD69, CD44, T-bet, IFN-γ, and TNF-α, but did not secrete IL-17A (Fig. 2, H and I). They also expressed high amounts of *Prf1* and *Gzmb* mRNA (Fig. 2 J). These findings confirm that CD8^+^ T cells exacerbate psoriasiform inflammation in the absence of T reg cells and exhibit an activated cytotoxic phenotype in the skin.

### Foxp3^+^ T reg cells control inflammation by restraining MNPs

Transcriptomic analysis revealed enrichment of IFN-I signaling in T reg cell–deficient inflamed skin (Fig. 1). This gene signature persisted when excess inflammation was reversed by CD8^+^ T cell depletion, suggesting a hierarchical superiority of IFN-I in driving excess inflammation in the absence of T reg cells. IFN-I are well-described drivers of CD8^+^ T eff responses and can be produced by a multitude of cells (McNab et al., 2015). We therefore sought to identify the source of IFN-I in T reg cell–depleted skin.

**Figure 3.**
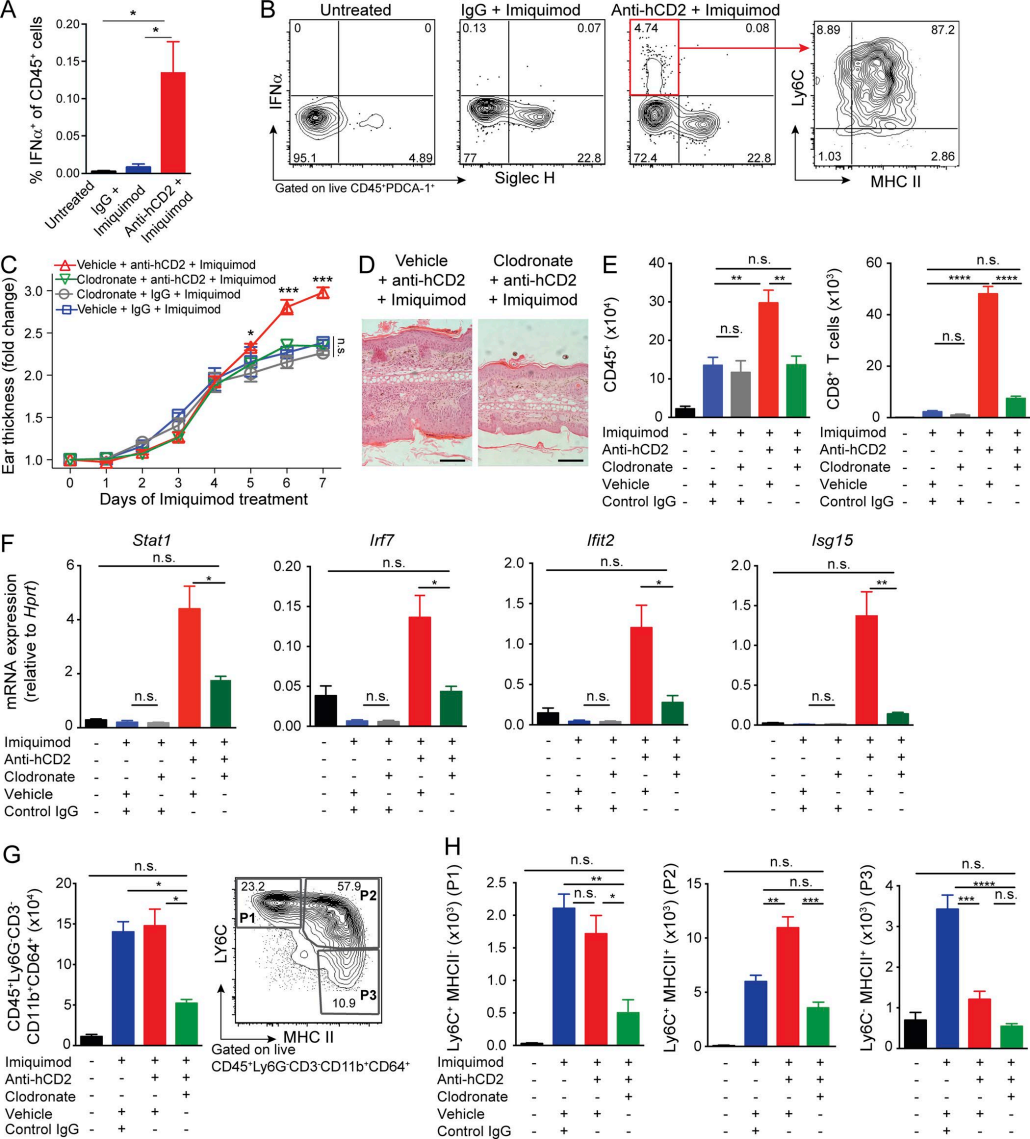
**T reg cells suppress IFN-I production by restraining MNPs. (A and B)** Flow cytometry analysis of CD45^+^ cells from cervical lymph nodes (stimulated with PMA/ionomycin for 4 h + BFA) of Foxp3^hCD2^ mice treated as indicated. **(A)** Percentage of IFN-α^+^ cells among CD45^+^ cells in cervical lymph nodes. **(B)** Surface marker expression of IFN-α^+^ cells pregated on CD45^+^PDCA1^+^ cells. **(C–F)** Depletion of MNPs using clodronate liposomes in mice treated with IMQ ± anti-hCD2. **(C and D)** Thickness of ear skin over the course of IMQ treatment (C) and representative H&E staining (D). Scale bars, 200 µm. **(E)** Quantification of skin CD45^+^ cells and CD8^+^ T cells by flow cytometry. **(F)** qPCR analysis of interferon response gene mRNA in skin tissue. **(G)** Total CD45^+^Ly6G^−^CD3^−^CD11b^+^CD64^+^ cells isolated from skin tissue assessed by flow cytometry (left) and MNP “waterfall" gating strategy P1–P3 (right). **(H)** Total cell numbers of P1–P3 assessed by flow cytometry. Error bars: means ± SEM. Statistics: one-way (A and E–H) and two-way ANOVA (C) with post-hoc test. Data are representative of one of three experiments with *n* ≥ 4 (C, D, G, and H) or one of two experiments with *n* ≥ 3 mice per group (A, B, E, and F). *P = 0.01–0.05, **P = 0.001–0.01, ***P = 0.0001–0.001, ****P < 0.0001, n.s., not significant.

Among CD45^+^ cells from cervical lymph nodes (which drain the ear skin), IFN-α was readily detected by flow cytometry only in cells from T reg cell–depleted, IMQ-treated mice (Fig. 3, A and B). The IFN-α^+^ population coexpressed MHCII, Ly6C, and PDCA1 (Fig. 3 B). Plasmacytoid dendritic cells (pDCs) are capable of secreting large amounts of IFN-I and were previously suggested to promote early psoriasis plaque formation in a xenotransplant model (Nestle et al., 2005). However, the pDC marker Siglec-H was not expressed by IFN-α–producing cells, identifying them as an MNP population (Fig. 3 B). Macrophages and monocyte-derived cells are capable of producing IFN-I and are found in higher abundance than pDCs (Ivashkiv and Donlin, 2014). Indeed, induction of IFN-I in skin macrophages has been described in *Leishmania major* infection and in cutaneous hypersensitivity reactions, where skin-resident, monocyte-derived cells and DCs showed high amounts of interferon-stimulated genes and a signature suggestive of IFN-I production (Diefenbach et al., 1998; Tamoutounour et al., 2013). Indeed, a recent study used single-cell analysis to demonstrate that human pDCs appear to be more closely related to classical DCs than previously considered, suggesting that functions generally ascribed to pDCs may also be features of additional myeloid populations (Villani et al., 2017).

To test the importance of MNPs in T reg cell–deficient, IMQ-treated mice, we depleted this population using clodronate-loaded liposomes (Van Rooijen and Hendrikx, 2010). Ear thickness measurements and skin histology demonstrated that MNP depletion prevented excess inflammation caused by combined IMQ treatment and T reg cell depletion (Fig. 3, C and D). Similarly, flow cytometry analysis of the skin showed that clodronate treatment reduced total CD45^+^ cells and CD8^+^ T cells in mice that received IMQ and T reg cell depletion to a level equivalent to that of T reg cell–sufficient, IMQ-treated animals (Fig. 3 E). Quantitative RT-PCR analysis of whole skin tissue confirmed that MNP depletion reduced the expression of IFN-I–regulated genes, demonstrating that MNPs play a nonredundant role in CD8^+^ T cell–mediated skin inflammation in T reg cell–deficient mice (Fig. 3 F). Clodronate did not affect IMQ-induced inflammation in T reg cell–sufficient animals in terms of ear thickness or amount of leukocytic infiltrate (Fig. 3, C, E, and F). As clodronate effectively reversed the T reg cell depletion effect, we sought to further characterize the responsible MNP population using a previously published flow cytometry strategy (Tamoutounour et al., 2013). CD45^+^LY6G^−^CD11b^+^CD64^+^ monocyte-derived cells were significantly reduced in the skin of clodronate-treated mice (Fig. 3 G). Further phenotyping of these cells based on Ly6C and MHCII expression revealed that they were predominantly LY6C^+^MHCII^+^ (Fig. 3 H). These CD45^+^LY6G^−^CD11b^+^CD64^+^Ly6C^+^MHCII^+^ cells were highly abundant and significantly increased in the inflamed skin of T reg cell–depleted animals and sensitive to clodronate depletion (Fig. 3, G and H), suggesting they may contribute to the IFN-I signaling, CD8^+^ T cell infiltration, and worsened inflammation observed in IMQ-treated, T reg cell–deficient mice.

### IFN-I mediates excess inflammation in the absence of Foxp3^+^ T reg cells

**Figure 4.**
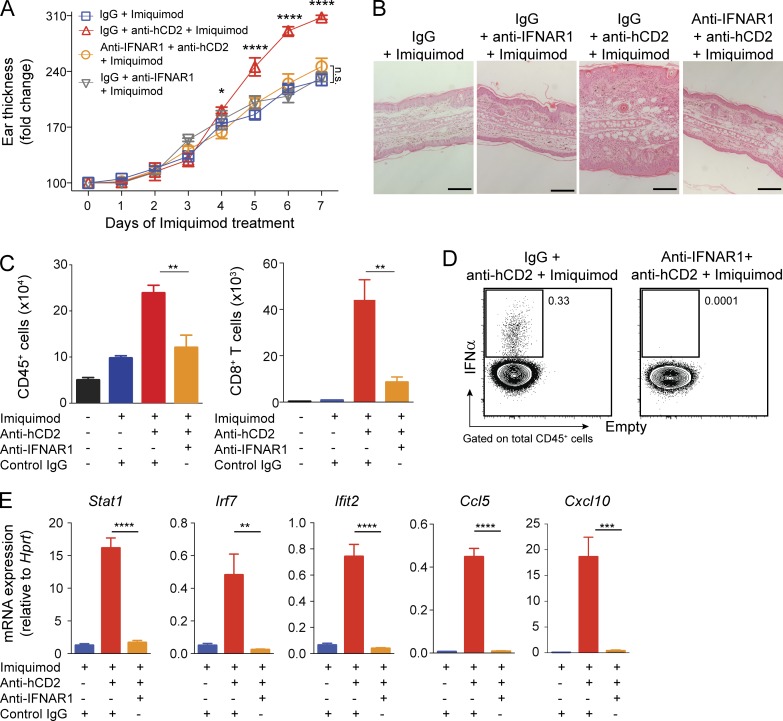
**T reg cells control skin inflammation by inhibiting IFN-I production. (A–E)** IFNAR1 blockade in mice treated with IMQ ± anti-hCD2. **(A)** Thickness of ear skin over the course of treatment. **(B)** Representative H&E-stained skin of treatment groups as indicated. Scale bars, 200 µm. **(C)** Quantification of total skin CD45^+^ cells and CD8^+^ T cells by flow cytometry. **(D)** Representative flow cytometry plot of total IFN-α^+^ cells in cervical lymph nodes at day 7 of IMQ treatment. **(E)** qPCR analysis of interferon response gene mRNA in skin tissue. Error bars: means ± SEM. Statistics: one-way (C and E) and two-way ANOVA (A) with post-hoc test. Data are representative of one of three experiments with *n* ≥ 4 (B and C) or one of two experiments with *n* ≥ 4 (A and E) or *n* ≥ 3 mice per group (D). *P = 0.01–0.05, **P = 0.001–0.01, ***P = 0.0001–0.001, ****P < 0.0001, n.s., not significant.

To establish whether IFN-I is required for the pathology of T reg cell–depleted mice, we administered an IFNAR1 (interferon α/β receptor α-chain)-blocking antibody to inhibit IFN-I signaling in T reg cell–sufficient and T reg cell–depleted, IMQ-treated mice (Fig. 4 A). Ear thickness, skin histology, and total numbers of skin-infiltrating CD45^+^ cells demonstrated that IFN-I signaling was required for increased pathology in T reg cell–depleted mice (Fig. 4, A–C). Anti-IFNAR1 treatment did not affect IMQ-induced inflammation in T reg cell–sufficient animals (Fig. 4, A and B). CD8^+^ T cells in the IMQ-treated, T reg cell–depleted skin were greatly reduced by IFNAR1 blockade (Fig. 4 C), suggesting that IFN-I signaling acts as an upstream driver of the T eff response elicited by T reg cell depletion. Furthermore, IFNAR1 blockade reduced the expression of IFN-α itself by cervical lymph node leukocytes (Fig. 4 D) and decreased the expression of interferon-stimulated genes in skin tissue to levels observed in T reg cell–sufficient skin (Fig. 4 E). Together, these findings demonstrate that T reg cell deficiency in IMQ-treated mice causes aberrant expression of IFN-I by MNPs, which drives a potent CD8^+^ T cell response that exacerbates skin inflammation.

In summary, we have identified a previously unappreciated role for T reg cells in controlling CD8^+^ T cells via the restraint of MNPs that secrete IFN-I during psoriasiform skin inflammation. Like other chronic autoimmune diseases, psoriasis is a long-term condition with phases of different disease activity (Perera et al., 2012; Lowes et al., 2014). Limitations of human tissue sampling and disease heterogeneity have hampered the study of plaque formation. T reg cells may play distinct roles at various time points in the inflammatory process, and our data suggest that they could control disease induction via their ability to restrain IFN-I production by MNPs.

The model used in our study involves a 7-d application of IMQ and is therefore a better model of acute plaque formation than it is of chronic disease. Our results are consistent with prior reports of plaque formation that described roles for IFN-I and CD8^+^ T cells (Nestle et al., 2005; Di Meglio et al., 2016), but reveal a previously unappreciated role for T reg cells in controlling that process. The approach used here could assist in experimental hypothesis testing at an early stage of disease that is difficult to study in patients.

Psoriasis pathogenesis is T cell dependent. However, the role of T reg cells in this process requires clarification. T reg cells have been suggested to acquire an unstable phenotype in psoriasis that promotes pathology (Soler and McCormick, 2011; Yang et al., 2016). The data presented in this study identify a novel role for T reg cells in controlling acute psoriasiform inflammation of the skin by negatively regulating IFN-I and CD8^+^ T eff cells. Epidermal CD8^+^ T cells are characteristic of human psoriasis, but this feature is rarely observed in murine models (Di Meglio and Duarte, 2013), suggesting that combined IMQ treatment and T reg cell depletion may more accurately reflect human pathology. Interestingly, cancer immunotherapies highlight the reciprocal relationship of regulatory pathways and skin CD8^+^ T cell responses. For example, T reg cells constitutively express CTLA-4, and *Ctla4^−/−^* mice closely resemble *Foxp3^−/−^* mice (Read et al., 2000; Takahashi et al., 2000). Anti–CTLA-4 immunotherapy (e.g., ipilimumab) can effectively treat malignancies such as melanoma but triggers immune-related adverse events that include skin reactions in up to 50% of patients. Vitiligo, a CD8^+^ T cell–mediated autoimmune disease, can be seen in these patients and is associated with favorable clinical response (Linardou and Gogas, 2016). Mogamulizumab, which targets the T reg cell skin-homing receptor CCR4, can cause a severe rash dominated by CD8^+^ T cells that similarly indicates good therapeutic response in cancer patients (Ishida et al., 2013; Yonekura et al., 2014; Kurose et al., 2015). Tissue-resident CD8^+^ T cells have a fundamental role in antiviral immunity and localize to skin and mucosal tissues, where they contribute to early host defense (Ariotti et al., 2014; Schenkel et al., 2014). It will be of interest to further explore the relationship between T reg cells and these tissue-resident T eff cells during infection of skin and mucosal tissues. CD4^+^ T cells also increased in IMQ-induced skin inflammation following T reg cell depletion. CD4^+^ T cells play an important role in supporting CD8^+^ cytotoxic T cell responses, and further experiments are required to establish their role in the CD8^+^ T cell–dependent, IFN-I–driven skin inflammation revealed by IMQ challenge in the absence of T reg cells (Sun and Bevan, 2003; Bevan, 2004). T reg cells have been demonstrated to promote IL-17/Th17 responses in mucosal infections (Pandiyan et al., 2011; Moore-Connors et al., 2013). It remains to be determined if T reg cells contribute to the γδ17 response observed in T reg cell–sufficient conditions.

In the present study, IFN-I signaling was a prominent feature of T reg cell–depleted inflamed skin. This was accompanied by an increase in IFN-α producing Ly6C^+^MHCII^+^ MNP in the lymph node. These cells also accumulated in inflamed skin, and their depletion by clodronate correlated with reduction of disease. Together with recently published data on wound healing, this supports an important role for T reg cells in controlling pro-inflammatory Ly6C^high^ populations in the skin (Nosbaum et al., 2016). Interestingly, the pathology elicited by IMQ treatment is largely equivalent in wild type and *Ifnar1^−/−^* mice, which appears surprising, as IMQ is a TLR7 agonist (Walter et al., 2013; Wohn et al., 2013). Our data help explain this finding by demonstrating that IFN-I signaling becomes pathological only when T reg cell function is compromised in IMQ-treated mice. Although IFN-I was necessary for aggravated pathology in T reg cell–depleted mice, the required IFNAR^+^ responder cells were not identified. This is challenging due to the plethora of IFN-responsive cell types but could feasibly be achieved using tissue- or cell-specific IFNAR1 deletion systems. In the present study, T reg cell depletion was achieved by systemic administration of anti-hCD2 antibody. Further exploration of the effects of targeted T reg cell depletion in skin could offer additional insight into spatial immune regulation. The precise mechanism by which T reg cells control IFN-I production by innate immune cells remains to be determined. However, T reg cells act via diverse regulatory mechanisms, and it is likely that several work in concert to control IFN-I production and skin inflammation (Campbell and Koch, 2011; Ali and Rosenblum, 2017).

Despite considerable advances in therapy of moderate to severe psoriasis with cytokine-targeting antibodies and other approaches, the pathophysiology of acute plaque formation and the contributing cell populations remain poorly understood, and thus difficult to target therapeutically. The present study provides new insight into this dynamic process and could assist further efforts to identify novel therapeutic approaches for early-stage disease.

## Materials and methods

### Mice

Wild type C57BL/6 (B6) and B6 Foxp3^hCD2^ mice (Kendal et al., 2011) were bred and maintained under specific pathogen-free conditions in accredited animal facilities at the University of Oxford. All experiments were conducted in accordance with the UK Scientific Procedures Act of 1986 and were performed by persons holding a personal license under a project license authorized by the UK Home Office. Mice were ≥7 wk of age when used for any experiments described.

### IMQ model and in vivo treatments

Cream containing 5% IMQ (Aldara; 3M Pharmaceuticals) was topically applied to the dorsal and ventral sides of both ears. Treatment was repeated with 20 mg of cream per ear every 24 h for 7 consecutive days. Mice were sacrificed 24 h after the last application. Ear thickness was measured before first application and daily before treatment with a dial thickness gauge (Mitutoyo UK). The mean of two measurements (one per ear) was determined. The following unconjugated monoclonal antibodies were administered in vivo as described in detail in every figure; control groups received suitable control IgG via the same route and at the same dose. To deplete Foxp3^+^ cells in B6 Foxp3^hCD2^, 250 µg anti-human CD2 (YTH 655) was administered in B6 Foxp3^hCD2^ mice i.v. on days −1 and 0, and i.p. on days 2, 4, and 6 of IMQ treatment. Anti-mouse CD8 antibody (53–6.72) was injected i.p. at 120 µg/dose on days −1 and 0, and on days 2, 4, and 6 of IMQ treatment. MNPs were depleted using clodronate liposomes. 0.2 ml of clodronate or PBS (control) liposomes were injected i.p. on days −1, 0, 2, 4, and 6 12 h after anti-hCD2 injection. To block IFN-I signaling, mice were treated with 1 mg of anti-mouse IFNAR1 antibody (MAR1-5A3) i.p. on day −1 of IMQ treatment.

### Tissue preparation

Ears were harvested at the base after sacrifice and stored on ice in PBS/BSA after collection of tissue for histological assessment and RNA extraction. Ears were mechanically split and finely minced. Ears were digested in RPMI with BSA, collagenase D (Roche), and Liberase TM (Roche) at 37°C for 80 min. Lymph nodes were mashed through a 70-µm mesh, washed in RPMI with BSA, and collected by centrifugation. Cell suspensions were filtered, centrifuged, and resuspended in PBS with BSA. Leukocytes from skin tissue were separated by centrifugation through Lymphoprep (StemCell Technologies, Inc.) and collected in appropriate buffer for further use.

### RNA extraction, cDNA synthesis, and quantitative PCR (qPCR)

Tissue was disrupted and cells lysed using a Polytron PT 1200 E manual disperser with a 7-mm dispersing aggregate (Kinematica) in RLT buffer (Qiagen). RNA was isolated using an RNeasy Mini kit (Qiagen) followed by RT using random primers (Applied Biosystems). qPCR was performed using TaqMan assays (Applied Biosystems) and PrecisionPlus Mastermix (Primer Design) on a ViiA7 384-well real-time PCR detection system (Applied Biosystems). We used the following assays: *Stat1* Mm00439531_m1, *Irf7* Mm00516793_g1, *Isg15* Mm01705338_s1, *Ifit2* Mm00492606_m1, *Ccl5* Mm01302427_m1, *Cxcl9* Mm00434946_m1, *Cxcl10* Mm00445235_m1, *Prf1* Mm00812512_m1, and *Gzmb* Mm00442834_m1. All expression levels were normalized to an internal house-keeping gene (*Hprt*) and calculated as 2 − (CT*_Hprt_* − CT*_gene_*). For the whole tissue microarray, tissue was prepared as described above, and RNA was extracted using the RNeasy Mini kit (Qiagen). RNA was assessed for quantity (NanoDrop) and quality (Agilent 2100 Bioanalyzer), hybridized to Illumina Wg6 v2.0 Expression Bead Chips (Illumina), washed, and scanned using a HiScan scanner (Illumina). Probe signal intensities were extracted using GenomeStudio software and further analyzed. Probes expressing significantly above background levels (detection P < 0.05) were used for downstream processing. Array signal intensities were background adjusted, transformed using the variance stabilizing transformation, and quantile normalized using Lumi (Du et al., 2008) from R/Bioconductor. Differential expression analysis was performed for each contrast using the empirical Bayes method implemented in LIMMA (Ritchie et al., 2015). Significance was defined as a Benjamini-Hochberg adjusted P value <0.05 and log2 fold change >1. Enriched GO biological processes were assessed in differentially abundant gene sets using a hypergeometric test implemented in the runGO.py script from the CGAT code collection (Sims et al., 2014), and terms were considered significant at a false discovery rate <0.05. Microarray data have been deposited in the ArrayExpress database at EMBL-EBI under accession no. E-MTAB-6873.

### Flow cytometry and cell sorting

Mouse cells were stained with combinations of the following monoclonal antibodies according to manufacturer protocols: CD3 (145-2C11), CD4 (GK1.5), CD8 (53–6.7), CD45 (30-F11), Ly6G (1A8), PDCA1 (JF05-1C2.4.1; eBio927), SiglecH (eBio440c), TCRβ (H57-597), TCRγδ (GL3), MHC II (M5/114.15.2), Foxp3 (FJK-16s), and IFN-α (RMMA-1). Foxp3 detection in all figures was performed with intranuclear staining using FJK-16s. Viable cells were identified using eFluor-780 fixable viability dye (eBioscience). Stained samples were acquired on FACS LSRFortessa and FACS LSRII flow cytometers (Becton Dickinson). Data were analyzed using FlowJo (Tree Star, Inc.). For intracellular cytokine staining, cells were restimulated with PMA (5 ng/ml; Sigma-Aldrich), ionomycin (500 ng/ml; Sigma-Aldrich), and 5 µg/ml brefeldin A (Sigma-Aldrich) for 4 h followed by surface staining. Cells were fixed with 2% formaldehyde (Merck) and stained for intracellular cytokines in permeabilization buffer containing 0.05% saponin (Sigma-Aldrich). For Foxp3 staining, surface staining was followed by fixation and permeabilization with the Foxp3 staining buffer kit (eBioscience) according to manufacturer instructions. Cell numbers were calculated by adding 10^4^ CountBright beads (Life Technologies) into each well before acquisition on a flow cytometer. Postacquisition analysis was performed using FlowJo software (Tree Star, Inc.).

### Histology and immunohistochemistry

Ear tissue was harvested and submerged in PBS containing 10% formaldehyde. Tissue was embedded, and 5-µm-thick sections were stained with H&E. Images were taken using a Zeiss AxioScope 2. For immunofluorescence, ear tissue was snap frozen in optimal cutting temperature compound, sectioned into 7-µm-thick sections, and mounted on glass slides. Sections were fixed in –20°C acetone for 10 min and washed in PBS. Blocking solution was added for 1 h followed by washing and addition of primary antibody solution overnight. Slides were washed and stained with secondary antibody before washing and the addition of HOECHST nuclear stain for 1 h. Slides were mounted with coverslips using ProLong Gold mounting medium and imaged with a Zeiss 510 MetaHead confocal microscope.

### Statistical analysis

Statistical analysis was performed as indicated in the respective figures. Statistical significance levels are indicated in figures as follows: *P = 0.01–0.05, **P = 0.001–0.01, ***P = 0.0001–0.001, and ****P < 0.0001.

### Online supplemental material

Fig. S1 shows spleen weight, *Il17* expression in skin tissue, and cytokine production of γδ T cells.

## Supplementary Material

Supplemental Materials (PDF)
