# Supplementary material for: Foxp3+ T reg cells control psoriasiform inflammation by restraining an IFN-I–driven CD8+ T cell response
Source: J Exp Med. 2018 Aug 6;215(8):1987–98. doi: 10.1084/jem.20172094 (PMC6080913; doi:10.1084/jem.20172094)
Supplement: Supplemental Materials (PDF) [file JEM_20172094_sm.pdf]

Supplemental material

Stockenhuber et al., https://doi.org/10.1084/jem.20172094

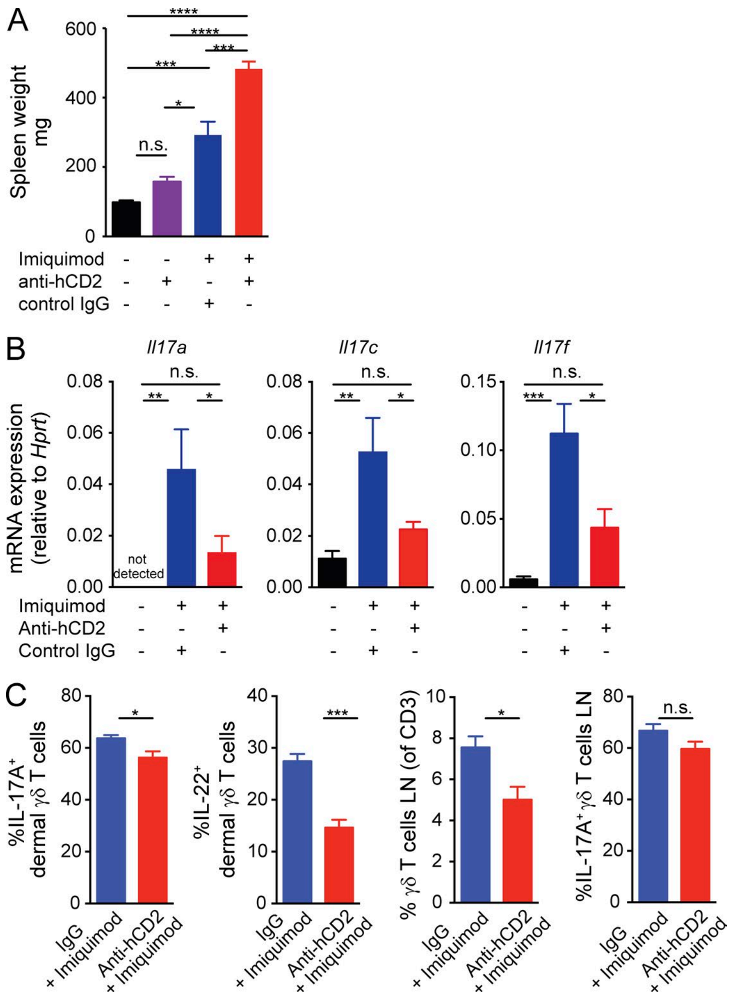

Figure S1. **Spleen weight, *Il17* expression in skin tissue, and cytokine production of γδ T cells. (A)** Spleen weight of untreated, anti-hCD2 only–, IgG + IMQ–, and anti-hCD2 + IMQ–treated mice. **(B)** qPCR analysis of mRNA isolated from skin. **(C)** Frequency of cytokine-producing dermal γδ T cells and cervical lymph node γδ T cells. Error bars: means ± SEM. Statistics: one-way ANOVA with post-hoc test (A and B) and Mann-Whitney *U* test (C). Data are representative of one of two experiments with $n \geq 4$ (A) and one of three experiments with $n \geq 3$ mice per group (B and C). *P = 0.01–0.05, **P = 0.001–0.01, ***P = 0.0001–0.001, ****P < 0.0001, n.s., not significant.
